# Morphological and Genetic Variation in Monocultures, Forestry Systems and Wild Populations of *Agave maximiliana* of Western Mexico: Implications for Its Conservation

**DOI:** 10.3389/fpls.2020.00817

**Published:** 2020-06-17

**Authors:** Dánae Cabrera-Toledo, Ofelia Vargas-Ponce, Sabina Ascencio-Ramírez, Luis Mario Valadez-Sandoval, Jessica Pérez-Alquicira, Judith Morales-Saavedra, Oassis F. Huerta-Galván

**Affiliations:** ^1^Laboratorio Nacional de Identificación y Caracterización Vegetal (LaniVeg-CONACYT), Departamento de Botánica y Zoología, Centro Universitario de Ciencias Biológicas y Agropecuarias, Universidad de Guadalajara, Zapopan, Mexico; ^2^Maestría en Ciencias en Recursos Naturales y Desarrollo Rural, Colegio de la Frontera Sur, Tapachula, Mexico; ^3^Cátedras CONACYT-Universidad de Guadalajara, Laboratorio Nacional de Identificación y Caracterización Vegetal (LaniVeg), Departamento de Botánica y Zoología, Centro Universitario de Ciencias Biológicas y Agropecuarias, Universidad de Guadalajara, Zapopan, Mexico; ^4^Maestría en Biosistemática y Manejo de Recursos Forestales y Agrícolas, Centro Universitario de Ciencias Biológicas y Agropecuarias, Universidad de Guadalajara, Zapopan, Mexico

**Keywords:** forestry systems, population genetics, morphological diversity, distilled beverages, raicilla

## Abstract

Forestry systems in Mexico are examples of traditional management of land and biodiversity that integrates the use, conservation and restoration of forest elements. Current *in situ* management practices of *Agave maximiliana* in western Mexico include the tolerance of many forest elements, reintroduction of young *Agave* plants and germination of seeds. More intense forms of management include monocultures, which are agroindustrialized systems developed in more recent times and characterized by the establishment of high densities of *A. maximiliana* plants in deforested areas and abandoned agricultural lands. We compared monocultures, forestry systems and wild populations (i.e., non/slightly-exploited forests) in order to evaluate whether these practices have had an effect on intraspecific morphological and genetic variation and divergence. We also tested whether divergence has a positive relationship with environmental and geographic distance. We analyzed 16 phenotypic traits in 17 populations of *A. maximiliana*, and 14 populations were further examined by amplifying 9 SSR loci. We employed multivariate methods and analyses of variance in phenotypic and genetic traits to test whether clusters and the percentage of variation contained in the managed and wild categories can be identified. Tests of isolation by environment (IBE) and distance (IBD) were performed to detect the magnitude of divergence explained by climatic and geographic variables. We found that forestry systems are effective as reservoirs of morphological and genetic diversity, since they maintain levels similar to those of wild populations. Moreover, the monocultures showed similar levels, reflecting their recent emergence. While the species showed high morphological diversity (IMD = 0.638, SE ± 0.07), it had low to intermediate genetic diversity (A = 2.37, H_*E*_ = 0.418). Similar morphological and genetic divergences were found among populations, but these were not correlated with each other in population pairs. Non-significant morphological differentiation was found among categories. Only IBE was significant in the genetic structure (β = 0.32, *p* = 0.007), while neither IBE nor IBD was detected in the morphological differentiation. We discuss the implications of these results in the context of the weaknesses and strengths of *A. maximiliana* in the face of the socio-ecological changes predicted for the study area in the short term.

## Introduction

Agaves have considerable cultural, economic and ecological importance in North America (Gentry, 1982). Around fifty three species of *Agave* L. are used for mescal production (Colunga-GarcíaMarín and Zizumbo-Villarreal, 2007; Torres et al., 2015b); however, most of the plants utilized are not cultivated but rather extracted from natural populations by the simple gathering of plants (*ca*. 37 spp., Torres et al., 2015b). Of these, 53 species used for mescal production, around 12 species of *Agave* are managed *in situ* (Torres et al., 2015b), i.e., a range of practices other than simple gathering is conducted. These populations managed *in situ* constitute valuable Forestry (FS) and Agroforestry (AFS) Systems maintained in Mexico. The terms FS and AFS refer to a land utilization type with several criteria of classification (Nair, 1985). In Mexico, these criteria are based on a traditional management of land and biodiversity that integrates the use, conservation and restoration of multiple useful species of domesticated and wild plants and animals for different purposes (Moreno-Calles et al., 2013). While AFS represent alternatives to the current problems relating to the management and conservation of biodiversity (Torres-García et al., 2019), *Agave* monocultures have had an impact on traditional ecological knowledge and agrobiodiversity, while also depending heavily on the use of toxic agrochemicals (Bowen and Gerritsen, 2007). These agroindustrial management forms are characterized by the establishment of high densities of a single species for one simple purpose in deforested areas and/or abandoned agricultural lands (Torres-García et al., 2019). Less than ten *Agave* species are maintained in monocultures (Torres et al., 2015b). Monocultures of *Agave tequilana* F.A.C. Weber Azul, used in the spirits industry for the production of tequila, have spread rapidly in western Mexico, particularly from the end of the 20th century up to the present day (Leclert et al., 2010).

Analyses based on genetic and phenotypic markers have revealed that differences between wild and managed plant populations depend on life history traits, geographic distribution and cultivation history (Lindsay et al., 2018), as well as on the forms and intensities of management. These constitute an extraordinary broad spectrum of expressions of management types (Casas et al., 1999, 2017; Otero-Arnaiz et al., 2005; Illsey et al., 2007; Blancas et al., 2009, 2013; Parra et al., 2010, 2012; Guillén et al., 2011; Cruse-Sanders et al., 2013; Contreras-Negrete et al., 2015; Torres et al., 2015a). On one hand, agave species with a dominance of vegetative reproduction, form the primary gene pools from which a high diversity of traditional landraces have been artificially selected (*A. angustifolia* Haw. and *A. rhodacantha* Trel., Vargas-Ponce et al., 2007, 2009; Rivera-Lugo et al., 2018; Trejo et al., 2018). Vegetative reproduction can also become dominant in managed populations, although it is not a common reproductive system in wild populations (*A. parryi* Engelm., Parker et al., 2010) or in their wild relatives (*A. hookeri* Jacobi, Figueredo-Urbina et al., 2017). This can, in turn, produce faster morphological divergences between wild and managed populations (Parker et al., 2010, 2014; Figueredo et al., 2014; Figueredo-Urbina et al., 2017). The constant reintroduction of wild germplasm to traditional landraces and high exchange of germplasm between farmers can maintain high levels of genetic diversity (*A. hookeri*, Figueredo-Urbina et al., 2017; *A. angustifolia*, *A. rhodacantha*, Vargas-Ponce et al., 2009), compared to the wild populations.

On the other hand, species that present sexual reproduction, found in wild populations or managed in FS and monocultures, have been studied to a lesser extent. While morphological divergences can be detected between wild and managed species (*A. inaequidens* K. Koch, *A. cupreata* Trel. & A. Berger, Figueredo et al., 2014; Figueredo-Urbina et al., 2017), the genetic divergences explained by management have been reported as null (*A. inaequidens*, *A. cupreata*, Figueredo-Urbina et al., 2017) or incipient (*A. potatorum* Zucc., Félix-Valdez et al., 2016). This reflects the fact that the human activities practiced in these systems are still governed by natural gene flow.

Wild and domesticated *Agave* spp. used for the production of spirits have paniculate inflorescences, a frequent nocturnal anther dehiscence and, in some cases, occur in geographic areas that overlap with those of the long-nosed bats (Arita, 1991). These aspects suggest the occurrence of chiropterophily (Molina-Freaner and Eguiarte, 2003; Rocha et al., 2006; Estrella-Ruiz, 2008; León, 2013). Indeed, their strong relationship with the bats could have driven the radiation of the genera and their close linages (Flores-Abreu et al., 2019). These bats present a foraging behavior over a wide geographical range (e.g., *Leptonycteris curasoae* Miller, 1900, *L. yerbabuenae*, Martinez and Villa, 1940, of distances of up to 100 km, Horner et al., 1998; Medellín et al., 2018) which is congruent with the low genetic differentiation presented by some *Agave* species used for the production of spirits, and presenting sexual reproduction, (e.g., *A. inaequidens*, *A. palmeri* Engelm., *A. cupreata*, Table 1). Since this dynamic maintains highly diverse gene pools, monocultures of sexually reproduced plants lose genetic diversity at a slower rate (see *A. inaquidens*, *A. cupreata*, wild vs. monocultures, Table 1) compared to those of vegetative propagation (see *A. angustifolia* vs. *A. tequilana*; *A. inaequidens* vs. *A. hookeri*, Table 1).

**TABLE 1:** Genetic diversity (SSRs) and population differentiation parameters, pollination vectors and reproductive system of *Agave* species.

Species	Management status	H_E_	A	F_ST_; Φ_ST_	R.S.	Poll.	VP	References
***Mezcalero Agave* species**								
*Agave parryi*	Wild Antropogenic	0.62 0.43	7.09 2.57	0.34	–	Bats, passerines	Yes	Parker et al., 2010
*A. parryi*	Wild Cultivated	0.73 0.62	8.59 4.36	–	–	Bats, passerines	–	Lindsay et al., 2018
*A. palmeri*	Wild	0.67	7.9	0.013	–	Bats, passerines	–	Lindsay et al., 2018
*A. inaequidens*	Wild Managed Cultivated	0.70 0.73 0.69	7.6 7.7 7.1	0.11	SI	Bats	No	Figueredo et al., 2015
*A. cupreata*	Wild Cultivated	0.51 0.48	4 3.2	0.07	–	Bats, bees	No	Figueredo-Urbina et al., 2017
*A. potatorum*	Wild Managed Nursery	0.87 0.72 0.69	–	0.36	SI	Bats	No	Félix-Valdez et al., 2016
*A. angustifolia*	Wild Cultivated	0.45 0.59	30 8	0.16 –	– –	Bats –	Yes	Trejo et al., 2018
*A. tequilana* “Azul”	Cultivated	0	–	–	–	–	Yes	Trejo et al., 2018
*A. tequilana “*Sigüin”	Cultivated	0.49	–	–	–	–	Yes	Trejo et al., 2018
*A. tequilana* “Chato”		0.43	–	–	–	–	Yes	Trejo et al., 2018
*A. rhodacantha*		0.49	–	–	–	–	Yes	Trejo et al., 2018
*A.maximiliana*	Wild Managed Cultivated	0.44 0.39 0.43	2.5 2.3 2.3	0.41 0.38 0.36	–	Bats	No	This study
**Not *mezcalero Agave* species**								
*Agave utahensis subsp. utahensis*	Wild	0.49	5	0.24	–	Bats, birds, bees, and hawkmoths	Yes	Byers et al., 2014
*A. hookeri*	Cultivated	0.40		0.28	VP	–	Yes	Figueredo et al., 2015

*H_E_, expected heterozygosity; A, number of alleles per locus; F_ST,_ genetic differentiation; Φ_ST,_ genetic structure; R.S., reproductive system; SI, self-incompatible; Poll., pollinators; VP, vegetative propagation.*

In this study, we explored the case of *Agave maximiliana* Baker growing in pine-oak forest in western Mexico. This is one of the *Agave* species used in Jalisco for the production of spirits other than tequila. The spirit known as “raicilla” forms part of the history in this region thanks to mining, an important activity during the colonial period, when raicilla was in high demand but also prohibited by the Spanish crown, along with all agave spirits at that time (Valenzuela-Zapata, 2015). The spirit is known as “raicilla de la sierra” (hereafter, “raicilla”) corresponding to a highland region, and distinguishable from “raicilla de la costa,” which is produced using other *Agave* species (*A. rhodacantha* and *A. angustifolia*) in the coastal region of Jalisco. The historical impact of this activity on *A. maximiliana* populations has never been evaluated, and no landraces or ecotypes have been documented.

**FIGURE 1:**
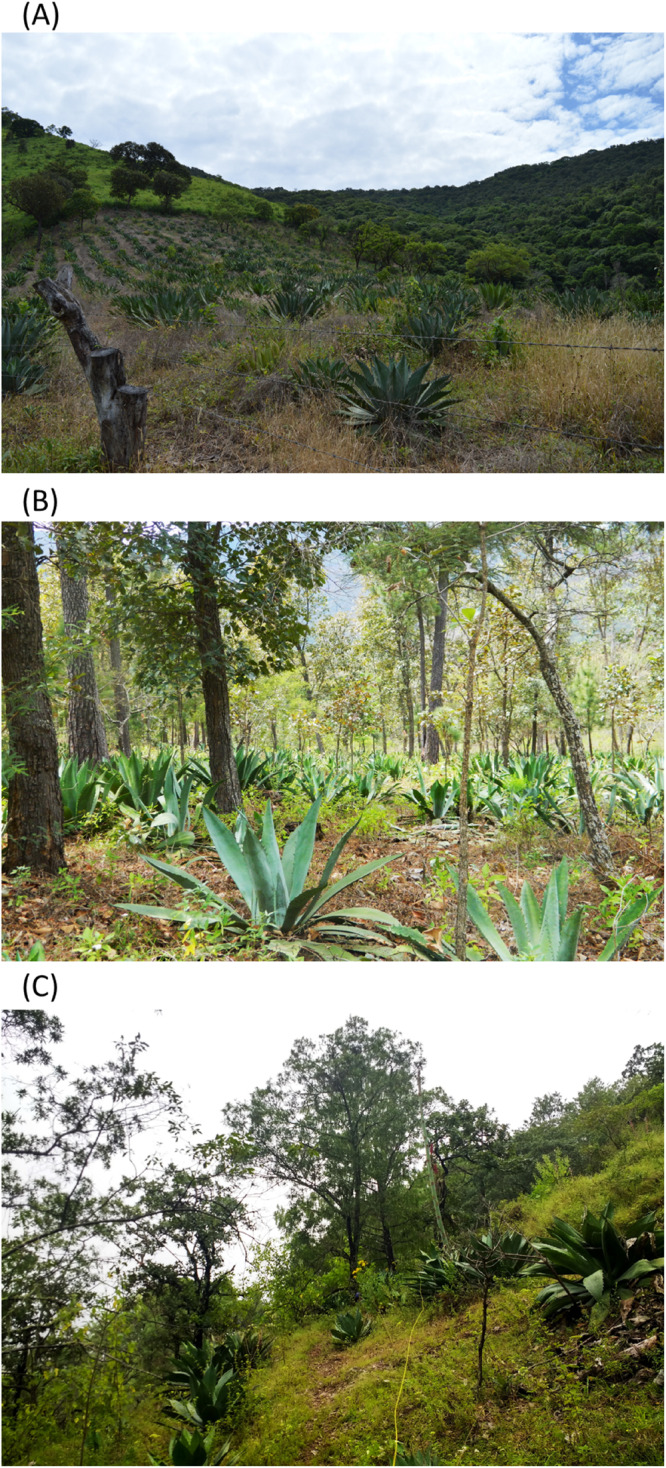
Categories of studied populations of *Agave maximiliana*. **(A)** Monoculture “El Mosco,” **(B)** Forestry System “Rincón de Mirandillas,” **(C)** Wild population “El Palmito.” Photos by: SA-R, DC-T and OH-G, respectively.

Recently, our research group carried out studies through structured interviews. The study showed that most of the people who produce raicilla have actually learned the tradition transversally; i.e., not as a result of inherited knowledge. It appears that there has been a resurgence of interest in this activity and that the management practices of this species are therefore probably recent (Huerta-Galván, 2018). In addition, the study showed that nearly 90% of people interviewed conduct “promotion,” a management practice that conserves many forest elements, such as pines and oaks that are maintained for the protection of the *Agave* plants against frost and pests. This action is a common element of *Agave* management in Mexico, especially on species managed at elevations of 800–2,500 m.a.s.l (e.g., *A. inaequidens*, *A. cupreata*), where frosts, are light and fleeting, but where some species such as *A. maximiliana* are frequently damaged (Gentry, 1982). The management of agave plants and native trees, both elements of the forest, are therefore combined in areas known as “forestry systems (FS)” which differ from AFS by the absence of domesticated crop elements (Nair, 1985; Torres-García et al., 2019). “Promotion” also includes the reintroduction of young plants (3 years of age) to the forest, by collecting seeds from multiple wild plants, the origin of which is located at a linear distance of less than 10 km, or buying plants from other producers. Most people also germinate seeds, the resulting seedlings of which are grown in their backyards and remove weeds from their FS or monocultures, either manually with tools or through the use of herbicides. Some people conduct simple gathering, where they establish closed seasons to allow population regeneration, and some few (nearly 30%) grow their plants in monocultures, which is a much more recent development over the last 15–30 years since the current boom in mezcal began (Huerta-Galván, 2018). The current FS therefore provide most of the raw material that sustains raicilla production in this temperate region. Monocultures are characterized by a high transformation of the habitat, with no associated native trees within these systems, and greater dependence on the use of agrochemicals. It is notable that there is no clear criteria regarding what traits are selected, except for one producer who selects large plants with the highest sugar content in the leaves. Management practice details and associated native tree species, where applicable, are presented in Supplementary Material SM1 for monocultures, forestry systems and wild populations. It is also important to mention that, while FS tolerate several tree elements naturally occurring in these habitats, a qualitative observation is that the density of shrub elements may be lower compared to that of wild populations. In contrast, the density of agave plants is generally higher in FS than in wild populations (Figure 1). While human actions are more evident in FS than in wild populations, it is important to mention that the latter are not pristine: they are found in accessible sites and present signs of light exploitation. Most of these populations present a small number of plants with the inflorescence removed, as these are occasionally sold as food.

A common perception among raicilla producers is that a drastic decrease (60–80%) has occurred in wild populations in the region in the last 30 years (stated by 60% of the informants in Huerta-Galván, 2018). If these perceptions reflect a true decrease, genetic bottlenecks and demographic stochasticity should be expected (Luikart and Cornuet, 1998; Frankham et al., 2006) in *A. maximiliana* populations. The plants are harvested prior to flowering, thus precluding the incorporation a high proportion of new individuals from one generation to the next (cf. Aguirre-Dugua and Eguiarte, 2013; Torres et al., 2015b). Moreover, the density of flowering plants is an important attractant for pollinators of *Agave* (Estrella-Ruiz, 2008) such that, even when some plants are allowed to flower, these may still be insufficient in number to maintain an effective pollination dynamic. Thus, overexploitation promotes population decline, leading to some cases of local extinction (e.g., *Agave potatorum*, Delgado-Lemus et al., 2014). There are no studies addressing the pollination system in *A. maximiliana* but, given the general aspects described above, i.e., paniculate inflorescences, probable nocturnal anthesis dehiscence and geographic area overlapping with that of *Leptonycterys yerbabuenae*, *L. nivalis* Saussure, 1860 (Arita, 1991) and *Choeronycteris mexicana* Tschudi, 1844 (Arroyo-Cabrales et al., 1982; Ortega-García et al., 2017), we infer a chiropterophilous syndrome, with these bat species as likely pollinators.

Finally, we envision social changes for the management of *A. maximiliana* in the short term, given that raicilla recently obtained its denomination of origin (DOR, Diario Oficial de la Federación, 2019). Considering the case of tequila production in the state of Jalisco, where an increased demand for plants to produce this spirit occurred following its DOR (Bowen and Valenzuela-Zapata, 2009; Leclert et al., 2010), we consider that a similar increase in the demand of plants for raicilla production is highly probable.

In summary, we consider that a recent but dynamic management exists in *A. maximiliana*, where increased population density of this species is a priority, along with the conservation of dominant forest elements, the benefits of which are still recognized by most of the producers; seeds and plants from multiple sites in the region are constantly reintroduced to forestry systems and monocultures and a high exchange of these germplasm also takes place among producers. These actions have acted to maintain, to date, the forestry systems that in turn support most raicilla production. Such systems conserve many elements of the original habitat and they are still governed, to some extent, by the environmental and geographical location effects of the natural landscapes. This is in contrast to the agricultural systems, i.e., monocultures, that present higher levels of habitat modification and more artificially controlled natural processes (e.g., pests, germplasm origin, density and reproduction of plants, etc.). Finally, we assume that the sexual reproductive system and probably chiropterophilous pollination system of *A. maximiliana* are biological strengths that have acted to maintain the connectivity of populations through gene flow.

In the context of these observations and assumptions, we aim to determine: (1) whether the methods and intensity of the management practices realized in *A. maximiliana* populations have had an effect on their intraspecific diversity; and (2) whether isolation by environment (IBE, Lichstein, 2007; Wang, 2013) and by distance (IBD) are significant in this species. We hypothesized that: (1) the recent management context will favor morphological and genetic diversity in this species, where divergence among categories is incipient, and thus we expect high morphological and genetic diversity for the species, with equal or higher levels in forestry systems compared to monocultures and wild populations and low differentiation among management categories; (2) while an assumed wide geographical range of the foraging behavior of the candidate pollinators of *A. maximiliana* will be reflected in a high genetic connectivity, the influence of geographical and environmental factors will have a positive relationship with the morphological and genetic divergences; therefore, we expect low genetic differentiation among populations and significant isolation by distance (IBD) and by environment (IBE). This is to say, the greater the geographical distance and the environmental differentiation, the greater the genetic and morphological divergence. Our purpose was to delimit and define the state of diversity and intraspecific differentiation of *A. maximiliana* growing in western Mexico in order to identify biological, ecological and management weaknesses and strengths in the face of imminent socioecological changes in the region.

## Materials and Methods

### Study Species

**FIGURE 2:**
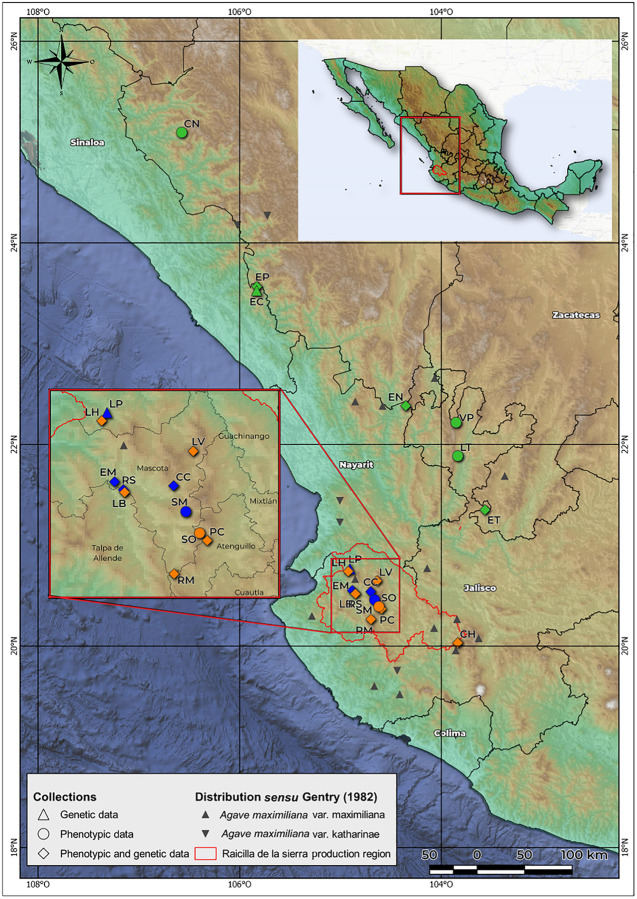
Geographical location of sampled populations. Green: wild populations. Orange: managed populations. Blue: cultivated populations. Raicilla de la sierra production region is the area in which raicilla is distilled and the *A. maximiliana* populations are apparently overexploited.

*Agave maximiliana*, known locally as “Lechuguilla,” is endemic to Mexico. It commonly inhabits rocky slopes in the states of Jalisco, Sinaloa, Durango, Nayarit, Colima and Zacatecas, at 900–2,000 m.a.s.l, and requires an annual precipitation of 750–1,000 mm (Gentry, 1982). It is frequent in pine-oak forests on calcareous soils or on those derived from igneous rock (Gentry, 1982; González-Elizondo et al., 2009). It is a perennial single rosette, monocarpic and medium size plant, balled or open, that habitually lacks asexual reproduction. Its leaves are broadly lanceolate, curved or straight, soft fleshy, with heteromorphic teeth of greater size toward the middle part of the leaf. The inflorescence is paniculate, of 5–8 m in height, branched, and with 15–30 small umbels. The greenish-yellow flowers are 52–65 mm in length. The fruits are small capsules of 3.5–5 × 1.7–2 cm, and are short oblong, stipitate, with rounded apex. The seeds measure 5.5–6 × 4.5–5 mm, the testa wavy, finely punctate, with marginal wing abruptly raised (Gentry, 1982; Vázquez-García et al., 2007). Gentry (1982) recognized two varieties with an overlapped distribution (Figure 2): *A. maximiliana* var. *maximiliana* and *A. maximiliana* var. *katherinae* (A. Berger) Gentry. The latter differs from the typical variety by its larger rosettes, greener leaves with more undulate repand margins with numerous variable interstitial teeth, and a deeper flower tube. However, McVaugh (1989) treated *A. maximiliana* var. *katherinae* as synonymous of *A. maximiliana sensu stricto*. Recently, Scheinvar (2018) defined the current potential distribution maps of both varieties and indicated that *A. maximiliana* var. *katherinae* could occupy the northern windward zone of the Sierra Madre Occidental (SMO), while *A. maximiliana* var. *maximiliana* could be found in the southern leeward zone of the SMO, but also in the Sierra Madre del Sur (SMS) and Transmexican Volcanic Belt (TVB) (Morrone et al., 2017). We perceived a wide morphological variation within populations in all sites sampled and decided to use *A. maximiliana* s.l. as recognized by McVaugh (1989).

### Study Area, Populations Sampled and Categorization

In accordance with our hypothesis, core populations of our study area were collected in western Jalisco in municipalities that produce raicilla (Figure 2). Populations sampled outside this area, in the states of Zacatecas, Nayarit, Sinaloa, and Durango, were considered wild populations (hereafter, “wild”) with little or no evidence of exploitation; these were used in the study as an external reference and for comparison of management gradients. Forestry systems (hereafter, “managed”) are those in which one or several of the practices described above are carried out (*sensu* Huerta-Galván, 2018). The monocultures (hereafter, “cultivated”) were established very recently, from around 30 or less years ago. The farmers use land in open areas that were previously used for other monocultures, usually maize. *Agave* rows are planted, almost always in lines following the slope. Agrochemicals are added to fertilize the plants or to eliminate weeds. Some farmers also apply chemicals on the leaves to combat pests. Monocultures have a high density of plants compared to plantations managed under the forest, and are characterized by a drastic change in land use. The seeds used for monocultures are collected from plants in the forest, domestic cultivation and *in vitro* propagation. This latter propagation type is currently carried out by a single producer only, who obtains 2–10 seedlings from each seed. This strategy is a response to the scarce availability of seeds and plants in recent years (Huerta-Galván, 2018).

Morphological data were obtained from 17 populations and the genetic analyses included microsatellite markers from 14 populations. For some populations, it was not possible either to obtain successful amplifications or to collect morphological data or leaf tissue (see Table 2). Finally, 12 populations were analyzed for environmental and geographic analyses using both genetic and morphological data.

**TABLE 2:** Populations of *Agave maximiliana* sampled for this study.

Population	Code	Data	State	Location	Elevation (m.a.s.l)
**Cultivated**					
Cimarrón Chico	CC	Phe/Gen	Jalisco	Mascota	1,519
El Mosco	EM	Phe/Gen	Jalisco	Mascota	1,181
Rincón Seco	RS	Phe/Gen	Jalisco	Mascota	1,267
San Miguel	SM	Phe	Jalisco	Mascota	1,599
Las Palmas	LP	Gen	Jalisco	San Sebastián del Oeste	1,000
**Managed**					
Chiquilistlán	CH	Phe/Gen	Jalisco	Chiquilistlán	1,938
La Berenjena	LB	Phe/Gen	Jalisco	Mascota	1,380
La Vieja	LV	Phe/Gen	Jalisco	Mascota	2,440
Los Hornos	LH	Phe/Gen	Jalisco	San Sebastián del Oeste	1,317
Puerto la Campana	PC	Phe/Gen	Jalisco	Atenguillo	1,968
Rincón de Mirandillas	RM	Phe/Gen	Jalisco	Mascota	1,630
Sol de Oros	SO	Phe	Jalisco	Mascota	1,876
**Wild**					
El Nayar	EN	Phe/Gen	Nayarit	El Nayar	2,269
El Palmito	EP	Phe/Gen	Sinaloa	Concordia	2,033
El Teúl	ET	Phe/Gen	Zacatecas	Teúl de González Ortega	1,699
Canelas	CN	Phe	Durango	Canelas	1,800
La Toma	LT	Phe	Jalisco	Bolaños	2,022
Valparaíso	VP	Phe	Zacatecas	Valparaíso	2,290
El Carrizo	EC	Gen	Sinaloa	Concordia	1,943

*Phe/Gen, Phenotypic and genetic data; Phe, phenotypic data; Gen, genetic data. m.a.s.l, meters above sea level. The localities are shown in Figure 2.*

### Data Analyses

To answer our research questions, the following general strategy was adopted: For testing the contribution of management to the diversity and differentiation of *A. maximiliana* populations, we first calculated levels of diversity in the morphological traits and tested for differences among categories. We then employed multivariate methods and permutational analysis of variance to test whether clusters and the percentage of variation contained among cultivated, managed and wild categories can be identified. Analogous methods were conducted for genetic traits where differentiation among populations was also important in terms of detecting the contribution of gene flow. Finally, to identify the contribution of geographical distribution and environmental factors to the morphological and genetic divergences, we determined the IBD and IBE.

### Diversity and Differentiation of Morphological Traits

Ten adult individuals were selected per population. Only plants with reproductive structures or visible indications of imminent reproductive maturity were selected, following the criteria of the producers. In total, 16 quantitative morphological traits were evaluated, as proposed by Vargas-Ponce et al. (2007) and Figueredo-Urbina et al. (2017) for agaves (Table 3). These traits are vegetative characters, since the presence of inflorescences is rare in cultivated and managed populations, and vegetative rather than floral characters are the targets of artificial selection. In each individual, three leaves of the mid verticil were measured in order to record variation and obtain mean values for these traits. Measurements were taken with a flexometer (resolution ± 0.001 m) and a ruler of 30 cm in length, and the data were used to construct a matrix for subsequent analysis. To discriminate the correlated morphological traits, a Pearson correlation test (*r* ≥ 0.9) was used following the criteria of Valdivia-Mares et al. (2016). Four traits (i.e., minimum diameter (MinD), leaf length (LL), leaf width at middle (LWm) and number of teeth in 10 cm^2^ (NT10) were excluded from the analysis because they presented multicollinearity (Table 3 and Supplementary Material SM2).

**TABLE 3:** Vegetative characters measured for wild, managed and cultivated populations of *Agave maximiliana*.

Variable	Code	P	Units
Total plant height	TPH	1	cm
Minimum diameter^a^	MinD*	1	cm
Maximum diameter	MxD*	1	cm
Leaf length^a^	LL	3	cm
Maximum leaf width	MxLW	3	cm
Leaf width at middle^a^	LWm	3	cm
Terminal thorn length	TTL	3	mm
Terminal thorn width at the base	TTW	3	mm
Number of teeth	NT	3	Amount
Number of teeth in 10 cm^2,a^	NT10	3	Amount
Longest tooth length	LTL	3	mm
Number of teeth/leaf length (thorniness)	NT/LL	3	Ratio
Number of teeth in 10 cm/leaf length (spacing)	NT10/LL	3	Ratio
Leaf width at middle/Maximum leaf width (width index)	LWm/MxLW	3	Ratio
Leaf length/Maximum leaf width (leaf shape)	LL/MxLW	3	Ratio
Terminal thorn width at the base/Terminal thorn length (thorn shape)	TTW/TTL	3	Ratio

P, Number of parts measured by individual; *two diameters were measured, in order to use the greatest one. ^a^Trait excluded from the morphological analysis due to high collinearity (see Supplementary Material SM2).

To analyze the morphological diversity per population and predicted management category, the Index of Morphological Diversity (IMD) was estimated. For this, the traits with the highest eigenvalues in the LDA (explained below) were selected in order to utilize those that better explained morphological diversity. Individual plant values of each trait from all populations were assigned to different morphological states according to the Sturges rule (Daniel, 2006). This procedure produced a matrix of discrete values and the IMD was estimated based on the Simpson Diversity Index (SDI). This index was defined as IMD =  1−Σ_1−*s*_(pi)^2^, in which pi is the proportion of the total number of individual plants sampled in a population showing the *i*th state of a morphological trait (categorized with the Sturges rule) and *s* is the number of states of that character (Casas et al., 2006). In this way, a unique estimator of the mean morphological diversity was obtained for all traits per population and category (cf. Figueredo-Urbina et al., 2017). The existence of significant differences among management categories by IMD was evaluated using a Kruskal Wallis test in Primer v6.1.11 (Anderson, 2017).

To estimate the multivariate differentiation among groups and determine which morphological traits have greater influence on the dispersion of the variation, a Linear Discriminant Analysis (LDA) was performed. For this, the database was standardized with the expression Y_0_ = (Y–*a*)/*b*); where Y_0_ is the standardized value, Y is the observed value of the state of a character, *a* is its mean and *b* is its standard deviation (Figueredo-Urbina et al., 2017). From the LDA, the centroids were obtained for each population, paired Euclidean distances were calculated and random resampling conducted to construct the null model (with 10,000 replicates) as a test of significance. To corroborate the identity of the cultivated plants, on the assumption that cultivated plants came mainly from managed rather than wild populations (if either the farmers collected seeds or bought the whole plant), we cross-validated the population assignation of each individual, from which we obtained a confidence value (i.e., the probability of correctly classifying an individual with respect to its population of origin). With these values and the predict function obtained from the LDA, we estimated the probable population of origin for each cultivated individual in monocultures (Legendre and Legendre, 1998; Medina-Villarreal and González-Astorga, 2016). All analyses were performed in R (R Development Core Team, 2012).

In order to determine whether the existing set of phenotypes differs significantly among management categories, a nested two-way permutational multivariate analysis of variance (PERMANOVA) was conducted with a type III (mixed effects) model: a fixed factor (condition) with three levels (wild, managed and cultivated) and a random factor (populations) with 17 levels. A PERMANOVA was performed with the records obtained from twelve non-correlated traits in Primer v6.1.11/PERMANOVA+ v1.0.1 (Anderson et al., 2008). First, a pretreatment was conducted in order to standardize the data (z-values) of the 12 traits analyzed, in order to transform the variables of different nature and measurement units to the same scale. A matrix of Euclidean distances was then constructed and the PERMANOVA performed. The statistical significance of the analysis was tested with 10,000 permutations based on a type III sum of squares. Likewise, the general patterns of morphological variation of the studied populations in each management category were analyzed using a Principal Coordinates Analysis (PCoA, Legendre and Legendre, 1998). This PCoA was performed based on the mean values per population of the 12 morphological traits used in the PERMANOVA. Therefore, the same pretreatment and Euclidian distance considered in the PERMANOVA design were used.

### Diversity and Differentiation of Genetic Traits

From each population, samples of plant tissue were collected from 15 individuals, transported at 4°C and stored at −45°C. From this tissue, DNA was extracted following Doyle (1991), modified with sodium chloride-tris-EDTA (STE). The DNA was quantified with a spectrophotometer and its quality evaluated by absorbance at 260/280 ng/μL. Dilutions at 40 ng/μL of all samples were prepared. A total of 16 microsatellites were tested; seven described by Lindsay et al. (2012), four by Parker et al. (2010) and five designed by Cabrera-Toledo (unpublished data) from the genome of *A. tequilana.* Only nine microsatellites were reproducible and polymorphic (Supplementary Material SM3). Amplification by PCR was conducted using the Multiplex PCR kit with TaqMan^®^ (Qiagen) in a final reaction volume of 11 μL. Each reaction contained 120 ng of DNA, 4 μL of Multiplex, 0.8 μL of each primer (10 mM) and 2.4 μL of molecular grade water (MiliQ). The amplification conditions were: 15 min at 95°C, followed by 40 cycles of 30 s at 94 °C, 30 s at the annealing temperature of each primer (Supplementary Material SM3) and an extension step of 2 min at 72°C, followed by a final extension step of 30 min at 60°C. The amplification was corroborated in 1.5% agarose gels with 1X TBE buffer. The PCR products of each primer per population were separated by vertical electrophoresis in 8% polyacrylamide gels (1–1.5 mm thickness) without urea. Each gel was left to run for 5 h. A 100 bp marker (Invitrogen) was used as a reference, and silver staining was used to visualize the bands (Sanguinetti et al., 1994). The gels were photo-documented with the software Gel Logic 100, Kodak and the fragment size in base pairs of the amplified bands was determined in Phoretix 1D v10 (Total lab Ltd.).

To analyze the genetic diversity, five parameters were evaluated: percentage of polymorphic loci (P), mean number of alleles per locus (A), mean number of effective alleles per locus (A_*E*_), observed heterozygosity (H_*O*_), expected heterozygosity (H_*E*_) under Hardy-Weinberg equilibrium (HWE). Also, we performed HWE analysis based on the *X*^2^ test (GenAlex 6.5, Peakall and Smouse, 2006). A Kruskal-Wallis test was conducted (SigmaPlot, v 13.0) to evaluate the significance of the differences in genetic diversity among management categories. The fixation indices F_*IS*_ and F_*IT*_ of Wright (1965) were used to evaluate inbreeding. To evaluate the possible effect of null alleles on the levels of inbreeding, a Bayesian Individual Inbreeding Model (IIM) analysis was performed in INest 2.2 (Chybicki and Burczyk, 2009). This permits non-biased calculation of the coefficient of inbreeding for multilocus data in the presence of null alleles (Fi). This analysis evaluates the effect of null alleles (*n*), inbreeding (*f*) and genotyping errors (*b*) on the values of homozygosity using the complete model (*nfb*) and its comparison with the model excluding the effect of the null alleles (*fb*), through the Deviance Information Criterion (DIC) between the two models. The analysis was run with 500,000 MCMC iterations and 50,000 burn-in cycles. Where the *nfb* model obtains the lowest DIC, this indicates that the null alleles have an important weight in the calculation of inbreeding. The null alleles also have a potential effect on genetic differentiation (F_*ST*_). To evaluate this, the FreeNA software was used to perform this estimation by detecting the frequency of null alleles for each locus and population based on the Expectation-Maximization (EM) algorithm (Dempster et al., 1977; Chapuis and Estoup, 2007). The population differentiation (F_*ST*_) was evaluated excluding null alleles, i.e., the ENA correction method (Chapuis and Estoup, 2007). A total of 50,000 bootstrap replicates were used to calculate the confidence intervals of the corrected global F_*ST*_.

Multivariate analyses were used to identify the genetic structure. First, analysis of molecular variance (AMOVA) was performed (GenAlex 6.5, Peakall and Smouse, 2012) to quantify the proportion of diversity within populations, among populations and among categories of management. This analysis uses the Φ_*ST*_ index, which is analogous to Wright’s index of differentiation F_*ST*_ (Hedrick, 2005). In addition, a Discriminant Principal Components Analysis (DPCA) (*Adegenet*, R Development Core Team, 2012) was executed to visualize the genetic structure in a graphic form. DPCA retains the virtues of a discriminant analysis, defining a model in which components of the genetic variation are maximized among groups and minimized within groups. The DPCA is supported by a transformation using a Principal Components Analysis (PCA) as a first step, guaranteeing that the subjected variables are not correlated and are lower in number than those of the individuals analyzed (Jombart et al., 2010). Finally, to visualize the degree of differentiation between pairs of populations, a paired matrix of genetic distances of Nei and Chakraborty (1973) was generated in the program GenAlex version 6.5 (Peakall and Smouse, 2012), with which a dendogram was constructed using the UPGMA method.

### Isolation by Distance/Isolation by Environment

#### Genetic/Morphological Distances – Geographic+Environmental Distances Matrix

To identify whether similar processes are molding morphological and genetic distances among populations, a simple regression analysis between these two distance matrices was conducted using the Multiple Regression Method (MRM) (Legendre et al., 1994; Lichstein, 2007) in R platform (ecodist v2.0.1, Goslee and Urban, 2007). The significance of the coefficient of regression was estimated with 1,000 permutations.

Separately, to test for IBD and IBE, we analyzed the relationship of genetic distances with geographic and environmental conditions, respectively, using the MRM method. The significance of the coefficient of regression was estimated with 1,000 permutations. The genetic distances were calculated by using the pairwise comparisons corrected for the presence of null alleles (Chapuis and Estoup, 2007) and linearized according to the equation F_ST_/1−F_ST_. The geographic distance matrix was generated using the geosphere package version 1.5.10 (Hijmans, 2015). To construct an environmental distance matrix, the 19 environmental variables of WorldClim Global Climate data version 2^1^, with a resolution of 30 arcsec (1 km^2^), were obtained. To reduce multicollinearity among environmental variables, those that presented a correlation ≥0.8 (JMP version 9, SAS Institute) were excluded. Separately, for each environmental variable, a simple regression analysis between the environmental distance matrix and the genetic distances was performed using the MRM method. In order to capture the most informative variables, those that exhibited a significant association with genetic distances were selected. Finally, Principal Component Analysis (PCA) was used to reduce the variation of these data, and the two first Principal Components (explaining 97% of the total variation) were used to calculate the Euclidean similarity distance matrix (PAST version 3.24, Hammer et al., 2001).

To test IBD and IBE in morphological distances, we performed the MRM method (1,000 permutations) using the geographical and environmental matrices generated with the procedure explained above. The morphological distance matrix was obtained based on the 12 morphological traits and the same pretreatment and Euclidian distance used in the PCoA and PERMANOVA (Primer v6.1.11.).

## Results

### Morphological and Genetic Diversity

The Index of Morphological Diversity (IMD) reflected a general mean of 0.638 ± 0.07 (interval 0.48–0.75, Supplementary Material SM4). In the cultivated populations, a narrow interval of 0.620–0.673 was observed. In the managed populations, this interval broadened to 0.493–0.747 and in the wild populations it was 0.460–0.700. However, no significant differences were found in mean IMD among categories or populations (H = 16, *p* = 0.453), suggesting that the plants were morphologically diverse regardless of management condition or the population to which they belong. However, variation tended to be lower among cultivated populations (SD ± 0.03), compared to the managed (SD ± 0.09) and wild populations (SD ± 0.1) (Supplementary Material SM4).

The mean percentage of polymorphic loci for all of the populations was 94.75%, the mean number of alleles per locus was 2.37 and the mean number of effective alleles was 1.87. There was an observed heterozygosity of 0.295 and an expected heterozygosity of 0.418 (Supplementary Material SM4). Mean values per management category (Table 4) did not present significant differences in any of the parameters of genetic diversity (P, A, A_*E*_, H_*O*_ and H_*E*_). On average, close to half of the loci (49.2%) were found to be in Hardy-Weinberg equilibrium, with slight variation presented in each category (cultivated 41.67%, managed 51.85% and wild 52.78%). Another 38.09% of the loci, on average, were found to present a deficit of heterozygotes, also with slight variation among categories (cultivated 38.89%, managed 35.18% and wild 41.67%). A total of 7.14% of the loci presented greater heterozygosity than expected in equilibrium: the highest average (13.89%) was found in the cultivated plants, followed by the managed (5.56%) and wild (2.78%) plants. The inbreeding coefficient was F_*IS*_ = 0.273 ± 0.134, only five of the nine loci were significant (estimated *X*^2^ > critical value, *p* = 0.001). The loci that did not present local inbreeding corresponded to the primers 12, 1763, 28 and 20. A frequency of null alleles was found in a range of 0–0.392. Inbreeding corrected for null alleles was of Avg (Fi) = 0.1529, with a density interval after 95% of 0.0024–0.3179. The model with the lowest DIC value was *nfb* (DIC: 10216), compared to the model *fb* (DIC: 10424), indicating that the null alleles had an important effect on the calculation of the inbreeding.

**TABLE 4:** Mean genetic and morphological diversity in populations of *A. maximiliana*.

Categories	N	NP	P	A	A_E_	H_O_	H_E_	IMD
Cultivated	12.44	4 (G,Ph)	94.44	2.30	1.89	0.359	0.427	0.648
	±0.82		±6.41	±0.14	±0.16	±0.08	±0.07	±0.03
Managed	12.7	6 (G), 7 (Ph)	92.59	2.33	1.82	0.248	0.388	0.674
	±1.13		±13.45	±0.29	±0.22	±0.07	±0.07	±0.09
Wild	12.5	4 (G), 6 (Ph)	97.22	2.5	1.92	0.277	0.438	0.591
	±1.07		±5.55	±0.23	±0.18	±0.10	±0.07	±0.10
Total	12.55	14 (G), 17 (Ph)	94.75	2.37	1.87	0.295	0.418	0.638
	±0.19		±2.54	±0.06	±0.05	±0.02	±0.02	±0.07

*N, sample size per population; NP, number of populations, G, genotypic data, Ph, phenotypic data; P, percentage of polymorphic loci; A, number of alleles per locus; A_E_, effective number of alleles per locus; H_O_, observed heterozygosity; H_E_, expected heterozygosity in Hardy-Weinberg equilibrium; IMD, index of morphological diversity ± standard error.*

### Morphological and Genetic Structure

**FIGURE 3:**
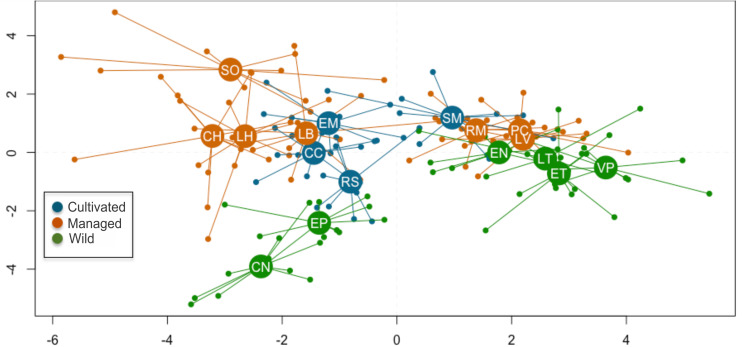
Scatterplot of discriminant analysis, explaining 70% of the total morphological variation. Small circles represent the 170 adult individuals evaluated, while larger circles are the centroids in each population. See Table 2 for definition of codes.

**FIGURE 4:**
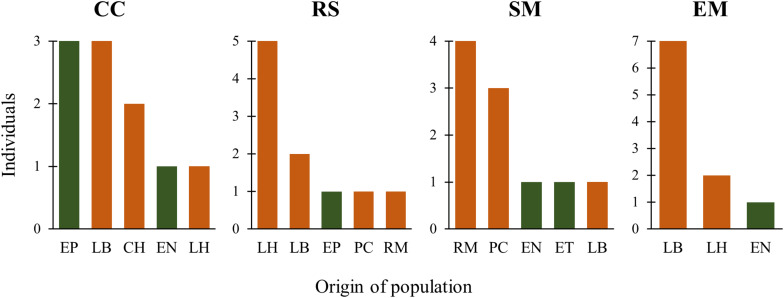
Histograms of probable origin of cultivated plants according to their morphological similarity. Green: wild populations, orange: managed populations. Black letters are monoculture codes. CC = Cimarrón Chico, RS = Rincón Seco, SM = San Miguel, EM = El Mosco. The names of the population of probable origin follow those in Table 2.

**FIGURE 5:**
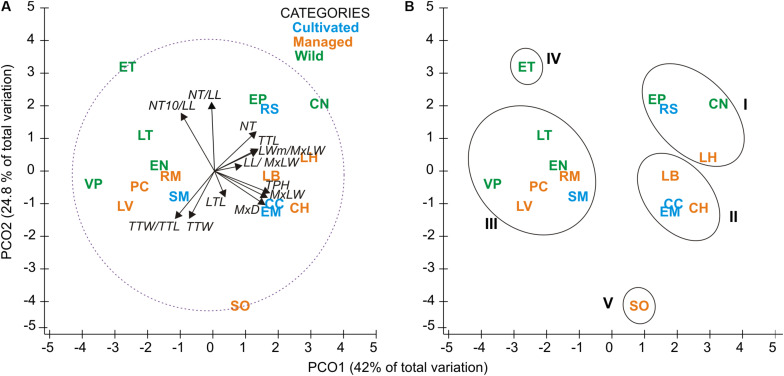
Principal coordinates analysis (PCoA) ordination; **(A)** Relationships among populations and morphological traits. Longitude of vectors corresponds to the magnitude of the contribution (correlation) of each trait, and the circle with dotted line represents the Pearson correlation ranges from -1 to 1. **(B)** Ellipsoids correspond to population groups (I to III) with similar traits, and two isolated entities (IV and V) with divergent traits. See Tables 2 and 3 for definition of codes.

The first two functions of the Linear Discriminant Analysis (LDA) explained 70% of the morphological variation. The maximum leaf width (MxLW) had the highest value in the LDA1 and traits related to plant thorniness (NT/LL; NT) and plant size (MxD) had the highest values in the LDA2. This analysis did not define a congruent grouping with the management categories established *a priori* (Figure 3 and Supplementary Material SM5). The cross validation test showed confidence values of between 30 and 100%, which corresponded to the proportion of individuals correctly identified in their population of origin based on morphological similarity. Prediction functions reclassified monocultures by their morphological similarity within mainly managed populations (Figure 4). Moreover, the PERMANOVA evidenced that 36% of the variation was presented among the populations (*p* < 0.05) with a non-significant 19.8% variation found among *a priori* categories (Table 5). Likewise, the PCoA showed a wide pattern of variation similar to that identified by the LDA and PERMANOVA. In PCoA (Figures 5A,B) three groups of populations and two isolated entities were distinguished. Group I comprised LH, EP, RS and CN, which are plants with a tendency to present high numbers of teeth (NT ≥ 73), large terminal thorns (TTL ≥ 32 mm) and more elliptic leaves (LWm/MxLW = 0.9) compared to the rest of the populations, which tend to have spathulate leaves (LWm/MxLW = 0.8). Group II comprised CH, CC, EM and LB, which include the largest and robust plants, of greatest height (TPH ≥ 116 cm), maximum diameter (MxD ≥ 176 cm), and with the widest leaves (LWm ≥ 19.5 cm). Group III comprised VP, EN, RM, SM, PC, LV and LT, which present plants of smaller dimensions (compared to group II) and most populations with a high thorniness index (NT10/LL = 0.2), as well as shorter and stouter terminal spines (TTW/TTL = 0.2). Finally, the ET and SO populations are isolated entities: ET (group IV) had the smallest plants (TPH = 58 cm; MxD = 101 cm; LWm = 10.8 cm) with the highest thorniness (NT10/LL = 0.4; NT/LL = 1.5) and, finally, SO (group V), much like group II, had large plants but presented the lowest thorniness index of all (NT10/LL = 0; NT/LL = 0.5) (Figures 5A,B). Even though such grouping tendencies can be observed, the values of the variation coefficient are wide in all traits (Supplementary Material SM6), constituting another expression of the high morphological variation of the species.

**TABLE 5:** Permutational analysis of variance (PERMANOVA) for twelve morphological traits in wild, cultivated, and managed populations of *A. maximiliana*.

Source	*df*	SS	MS	Pse-F	*p*-value	Unique permutations	% CV
Among categories	2	232.25	116.12	2.2255	0.0551	9916	19.80
Populations (categories)	14	730.49	52.178	7.4941	0.0001	9816	36.40
Residual	153	1065.30	6.962	–	–	–	45.18
Total	169	2028	–	–	–	–	–

*df, degrees of freedom; SS, sum of squares; MS, mean squares; Pse-F, Pseudo-F statistic; % CV, percentage of the components of the variation.*

**FIGURE 6:**
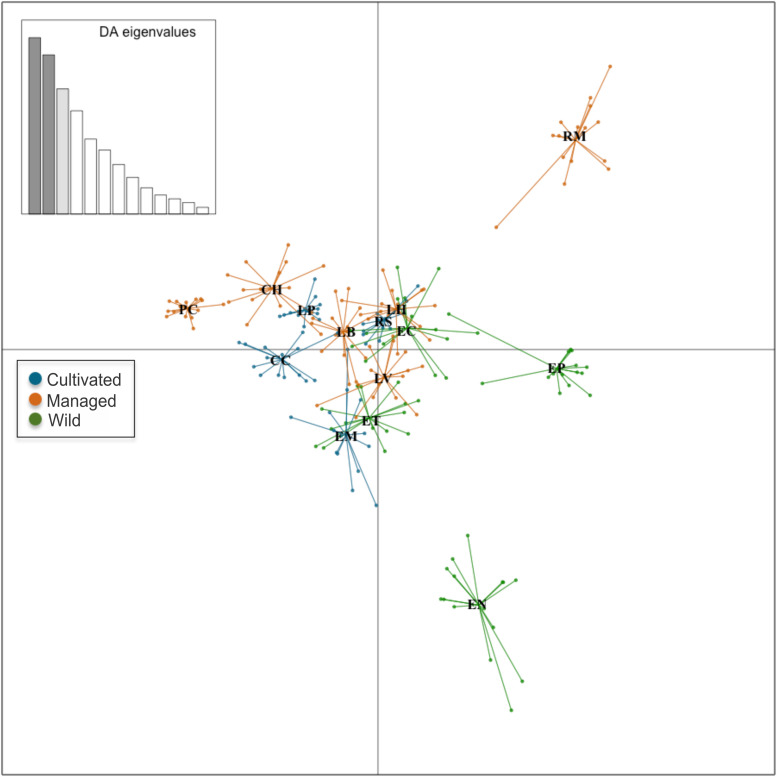
Discriminant analysis of principal components in nine loci and 14 populations of *A. maximiliana*. Small circles represent the 229 genetic samples. Lines represent the genetic variation within populations.

**FIGURE 7:**
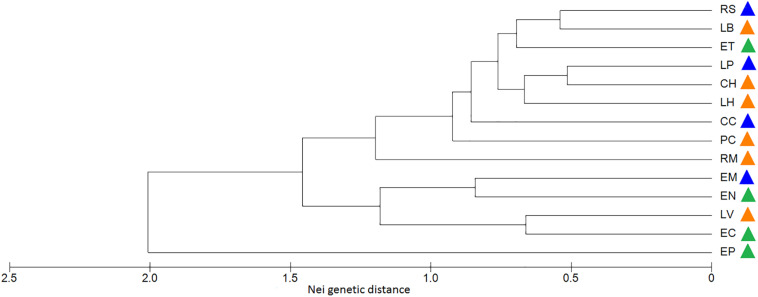
Dendrogram of genetic similarities in *A. maximiliana* populations based on Nei genetic distances. Genetically closer populations show the origin of clusters skewed to the right by reference of the Nei genetic distance scale. Green: wild; orange: managed; blue: cultivated. See Table 2 for definition of codes.

With respect to the genetic data, the AMOVA (Table 6) revealed that 61% of the variation was found within populations and 38% was found among populations (Φ_*CT*_ = 0.38, *p* = 0.001), which is similar to that found with the morphological data, while 1% corresponded to the variation among management categories (Φ_*PC*_ = 0.01, *p* = 0.003). Population differentiation, corrected for null alleles, was F_*ST*_ = 0.43 with ± 0.34–0.51 confidence intervals. Finally, the DAPC showed no genetic structure congruent with the pre-established categories (Figure 6). Most of the populations overlapped in the multidimensional space, apart from three: two wild (EN and EP), which were the most geographically distant, being located in Nayarit and Sinaloa, and one managed (RM). The population EC is geographically very close to EP (Figure 2); however, it overlapped genetically with managed and cultivated populations (Figure 6). The UPGMA showed a pattern of genetic distances that is not congruent with geography, but which groups together almost all of the cultivated and managed populations (except for EM and LV) (Figure 7). The wild EP is the most distant population of a second cluster group comprising a cultivated (EM) and a managed population (LV) from Jalisco, and two wild populations (EN) from Nayarit and (EC) from Sinaloa.

**TABLE 6:** Molecular analysis of variance based on 9 SSR loci, in wild, cultivated and managed populations of *A. maximiliana.*

Source	*df*	SS	MS	SV	Φ	*p*	% CV
Among categories (CT)	2	178.554	89.277	0.128	0.01	0.003	1
Among populations (PC)	11	886.710	80.610	4.876	0.38	0.001	38
Within populations	195	1521.257	7.801	7.801	–	0.001	61
Total	208	2586.522	–	12.806	–	–	100

*Φ_CT_ indicates the structure among categories; Φ_PC_ indicates the structure among populations within categories (F_ST_ analogous), and their respective percentage of variation (%) explained by the model. df, degrees of freedom; SS, sum of squares; MS, mean squares; SV, standard variation; Φ, Phi statistics; p, p value; % CV, percentage of the components of the variation.*

### Isolation by Distance/Isolation by Environment for Genetic and Morphological Distance Matrices

Morphological and genetic distances were not related to each other (*R*^2^ = 0.02, *p* = 0.22). The general model for IBD and IBE in terms of genetic data was not significant (*R*^2^ = 0.20, *p* = 0.06); however, we detected a significant effect of environmental variables on the genetic distance matrix (*β* = 0.32, *p* = 0.007). These variables were annual precipitation (BIO12; 785–1312 mm), precipitation of wettest month (BIO13; 187–333 mm), precipitation of driest month (BIO14; 2–11 mm), precipitation seasonality (BIO15; 87–115 mm), precipitation of warmest quarter (BIO18; 239–795 mm), precipitation of coldest quarter (BIO19; 45–156 mm). This result supports an IBE process, while the geographical distances matrix did not present a significant association with the genetic distances matrix (*β* = −0.13, *p* = 0.28) and therefore does not support an IBD process.

On the other hand, the MRM general model used to explore the influence of geographical and environmental variables on the morphological distance matrix was not statistically significant (*R*^2^ = 0.20, *p* = 0.06), and neither were the regression coefficients for the geographic and environmental distance matrices (*β* = 0.03, *p* = 0.74 and *β* = 0.13, *p* = 0.12, respectively).

## Discussion

This research showed that forestry systems of *Agave maximiliana* are effective as reservoirs of morphological and genetic diversity, since their levels are similar to those presented in wild populations. This result is congruent with other *Agave* species with similar management practices (*A. inaequidens*, *A. cupreata*, *A. potatorum*, Table 1). However, despite having similar reproductive and pollination systems to these species, *A. maximiliana* showed high levels of morphological diversity while, contrary to our hypothesis, the species presented low levels of genetic variation and high genetic differentiation among populations. Morphological divergences are not congruent with management categorization, although a slight genetic differentiation is. Morphological and genetic distances are not correlated, which suggests that different ecological-evolutionary processes are governing these traits. Alternatively, the number of loci evaluated might be not enough to test an association of genetic and morphological traits. Thus, further studies, including more genetic markers, are needed to analyze this relationship. Precipitation variables influence the environmental heterogeneity that in turn partly governs genetic structure in *A. maximiliana*. At the same time, no isolation by distance or by environment was detected in terms of the morphological differentiation in population pairs.

### Morphological and Genetic Diversity

Levels of morphological diversity in *A. maximiliana* are high (IMD = 0.628 on average, with an interval of 0.460–0.747), with values that are comparable to those reported for columnar cacti under *in situ* management categories, with mainly sexual (*Myrtillocactus schenckii* (J.A. Purpuss) Britton & Rose, IMD 0.652–0.703, Blancas et al., 2009) and, to a lesser extent, asexual (*Stenocereus pruinosus* (Otto ex Pfeiff.) Buxb. IMD 0.647–0.677, Parra et al., 2012) reproduction, but higher than *Stenocereus stellatus* (Pfeiff.) Riccob. with asexual reproduction (IMD 0.408–0.479, Casas et al., 2006). With respect to other agaves, the levels of morphological diversity are higher than those reported for species propagated from seeds (IMD = 0.413 *Agave inaequidens*; 0.489 *A. cupreata*) and from shoots (0.481 *A. hookeri*, Figueredo-Urbina et al., 2017). In all of the cases mentioned, managed populations present morphological diversity that is greater than or similar to that of wild populations. This provides evidence that traditional *in situ* management maintains the levels of diversity found in the wild state, and is in contrast to the forms of modern plant improvement responsible for the reduction of genetic and morphological diversity in global agriculture (Vellvé, 1992; Clunies-Ross, 1995). The evidence provided here confers *A. maximiliana* with the highest morphological diversity reported for a species of *Agave* to date.

In contrast, the low genetic diversity found in *A. maximiliana*, with similar values across the management categories (Table 4), suggests that there is a general biological and/or ecological process underway that is independent of the management context pre-established in this study. This result is incongruent with its life history characteristics, particularly the fact that it propagates from seed. The genetic diversity of *A. maximiliana* is lower than that of other long-living perennial cross-pollinating species (H_*E*_ = 0.61–0.68, Nybom, 2004), as well as that of its congeners *A. inaequidens* and *A. potatorum* (Table 1). It is barely comparable with other agaves of vegetative propagation (e.g., cultivated plants of *A. parryi*, *A. angustifolia*) and even equal or inferior to some landraces of domesticated species such as *A. angustifolia*, *A. rhodacantha* and *A. tequilana* (Table 1). Moreover, the levels of inbreeding found are significant at population level (F_*IS*_ = 0.273), although it should be noted that the inbreeding corrected for null alleles was lower (Fi = 0.153). This value must therefore be treated with some caution. Assuming that *A. maximiliana* has an outcrossing reproductive system, this would depend to a large extent on the pollinators. The low genetic diversity and presence of inbreeding in this species could be the consequence of low visit rates or inefficient pollinator behavior.

The Crenatae group, to which *A. maximiliana* (Gentry, 1982) belongs, includes species with strong inbreeding depression and a xenogamic pollination system that are pollinated by bats. In general, agaves with paniculate flowers are often pollinated by bats (Gentry, 1982; Rocha et al., 2006). However, the specific reproductive and pollination system of *A. maximiliana* has not been examined in detail. Arizaga et al. (2000) demonstrated in *A. macroacantha* Zucc. that, without pollination by bats, the plants produce only approximately 50% of the number of seeds. The size of the population of chiropterans that visit the plants influences the proportion of viable seeds (Howell and Roth, 1981; Arizaga et al., 2000). González (2005) found that visits by a low density of bats in *A. garciae-mendozae* Galván and Hernández produce low dispersion of pollen, since these bats visit flowers of a given plant at different stages, thus causing inbreeding.

Seed scarcity is frequently mentioned by various producers in the raicilla de la sierra region, and has led to the implementation of an *in vitro* reproduction technique from seeds in the local technical high school (pers. obs.). In some of the sample populations, there is also a high incidence of plants with poorly developed inflorescences; i.e., of small size and with few flowers, and with evidence of infection by fungi and bacteria, (pers. obs.). Low levels of genetic diversity are generally associated with inbreeding and this, in turn, can trigger a reduction in the average adaptation of the population, in a process known as inbreeding depression (Charlesworth and Charlesworth, 1987; Reed and Frankham, 2003; Hedrick, 2005). This association between genetic diversity and low seed production has been reported in congeners such as *Agave murphyi* Gibson (Gentry, 1982; Parker et al., 2007) and *Agave schottii* Engelm (Trame et al., 1995). Considering these arguments, inbreeding depression in *A. maximiliana* is an issue that merits further exploration in future studies.

It is notable that management has not eroded the genetic diversity of cultivated and managed populations and that these remain similar to that of the wild populations. This could reflect very recent processes of artificial selection, an active exchange of seeds and plants among farmers or a low intensity of exploitation that still does not genetically impact the populations (Casas et al., 2006; Parra et al., 2010). The maintenance and increase in local diversity of *in situ* managed and cultivated populations is relatively common in perennial plants or those with wide generation times in other regions of Mexico and America (*Stenocereus pruinosus*, Parra et al., 2010; *S. stellatus*, Cruse-Sanders et al., 2013; *Myrtillocactus schenckii*, Blancas et al., 2009; *Spondias tuberosa* Arruda, Neto et al., 2013; *Pouteria sapota* (Jacq.) H. E. Moore and Stearn, Arias et al., 2015). In Mexico, this is due to the broad spectrum of activities of *in situ* management that not only include the extraction of plants, but also manual dispersion of seeds, tolerance of some individuals and the protection and promotion of plants with characteristics that are desired by humans, among others. These are practices that have been widely documented for agaves in central (Torres et al., 2015a) and western Mexico (Vargas-Ponce et al., 2007; Huerta-Galván, 2018) and for other useful plants in the Tehuacan Valley region (Blancas et al., 2010).

### Morphological and Genetic Structure

Divergences in morphological and genetic characters evaluated for *Agave maximiliana* in the studied area respond mainly to ecological-evolutionary processes related to the habitat and, to a lesser extent, to management practices. This is evidenced by the wide dispersion of variation in morphological and genetic traits that does not correspond to the management categories established *a priori* and contrasts with that reported for congeners such as *A. parryi* (Parker et al., 2014), *A. inaequidens* and *A. cupreata* (Figueredo-Urbina et al., 2017), as well as other species in more advanced stages of domestication, such as *A. angustifolia* and *A. rhodacantha* (Vargas-Ponce et al., 2007, 2009). In these domesticated species, the phenotypic traits are adjusted to the assigned categories or the cultivars differ morphologically among themselves. Our results validate the information recorded by Huerta-Galván (2018), that the interchange of plants or seeds in monocultures of *A. maximiliana* came mainly from the forestry systems within the raicilla de la sierra region and that the monocultures themselves are very recent systems. This can be inferred from their morphological similarity, where 60% (CC) to 90% (EM, RS) of individuals in monocultures can be morphologically identified as belonging to other *in situ* managed populations (Figure 4). This can also be inferred by genetic similarity, where most of the monocultures and managed populations (except for EM and LV) tended to form a cluster (Figure 7). However, morphological and genetic variation is wider among populations (36 and 38%, respectively) than among categories (19.8 and 1%, respectively). Consequently, our study did not find significant divergence related to management.

Different factors are involved in morphological divergences compared to genetic structure, since these were not related to each other. A correlation between phenotypic variation and neutral genetic markers may occur if the same neutral processes (e.g., gene flow and genetic drift) have historically affected the spatial structure of both types of variation (e.g., Hipp et al., 2018; Rodríguez-Gómez et al., 2018). Another aspect is that populations usually exhibit contrasting spatial patterns for different genetic markers because the mutation rates, combined with the evolutionary forces acting on them, are not the same (Habel et al., 2015). It is therefore necessary to include more genetic markers to support these results. On the other hand, isolation by environment (six variables related to precipitation; *β* = 0.32, *p* = 0.007) and not geographic distance is one of the mechanisms that structure the populations of *A. maximiliana*. This means that, to some extent, populations are genetically isolated as a result of different precipitation conditions (e.g., annual precipitation varies from 785 to1312 mm, precipitation of warmest quarter varies from 239 to 795 mm; cf. Trejo et al., 2016). Local precipitation is related to the regeneration and colonization of plant populations (Jiang et al., 2019). Thus, the influence of precipitation on demographic dynamics may explain its effect on the population genetic structure as revealed by neutral markers (Jiang et al., 2019), where demographic parameters mediate ecological–evolutionary interactions via adaptive and non-adaptive mechanisms (Lowe et al., 2017). Gene flow could be reduced by the poor establishment success of immigrant seeds. Moreover, a decreased chance of outcrossing conditions may compound these gene flow reductions and accelerate the genetic fixation rate in populations (Jiang et al., 2019). This phenomenon not only appears in adaptive loci but could also extend to the whole genome via genetic drift caused by selective sweeps (Nosil et al., 2009). Since morphological variation is not explained by any of these isolation processes, this could be a sign to discard phenotypic plasticity (cf. Rodríguez-Gómez et al., 2018) in the evaluated traits. However, other approaches, such as wide genome scans or/and common garden experiments, are necessary to confirm these interpretations (de Villemereuil et al., 2018). Finally, it is important to mention that Scheinvar et al. (2017) modeled environmental niche changes in *A. maximiliana* and other congeners, and found that *A. maximiliana* could have undergone an extension of its environmental niche during the Last Interglacial period (LIG, 130 Kyr), followed by a reduction in the Last Glacial Maximum period (LGM, 31Kyr). Thus, past extension-contraction cycles in the demographic history of *A. maximiliana* could result in decreases in the levels of genetic diversity and increases in genetic structure (e.g., *A. lechuguilla* Torr., Scheinvar et al., 2017). This hypothesis should also be tested using phylogeographic approaches and with other types of molecular markers in order to support the main findings of this study.

The hypothetical presence of two varieties of *A. maximiliana* was not clearly reflected in our data. The two northern populations collected for this study (CN and EP, Figure 2) occupy a region in which *A. maximiliana* var. *katherinae* is probably distributed (Gentry, 1982; Scheinvar, 2018). Both populations are morphologically similar or close in the multivariate space (Figures 5A,B) to LH and RS, which are not characterized by larger rosettes or greater thorniness, compared to *A. maximiliana* var. *maximiliana* as Gentry (1982) described it. The population EP is differentiated genetically (Figures 6, 7) but we did not obtain a sufficient number of microsatellite amplifications from CN, and therefore do not know its genetic components and cannot determine its location in the genetic multivariate space. The population EC, which is geographically close to EP and also within the hypothetical distribution area of *A. maximiliana* var. *katherinae*, is grouped as a genetic entity similar to managed and cultivated populations of the raicilla de la sierra region. This suggests that an important gene flow is still perceptible between the two hypothetical varieties. We consider that further research with a sample design focused on this taxonomic approach is necessary to test this hypothesis.

Finally, *A. maximiliana* maintains the highest genetic structure reported to date among species of *Agave* (Φ_*ST*_ = 0.4, Table 1; Eguiarte et al., 2013). This is perhaps a reflection of the type and extension of the distribution areas of their populations; this can be perceived in other congeners, in which a discontinuous distribution is associated with high genetic structure, in contrast to those of continuous distribution (e.g., *A. parryi* vs. *A. palmeri*, Lindsay et al., 2018). Furthermore, together with the results of genetic diversity and inbreeding, this reinforces the need to investigate the reproductive and pollination system of this species. The higher the genetic structure, the greater the probability that populations will reflect changes as independent evolutionary units (Slatkin, 1994), whether through genetic drift or selection.

### Perspectives and Recommendations

Based on the evidence presented in this study, we consider that traditional management represented by forestry systems of *A. maximiliana* has been a sustainable option in terms of genetic germplasm conservation. However, the current threatening scenario of the use of *Agave maximiliana* as a raw material for agroindustrial production of raicilla is disheartening. Of particular concern are the low genetic diversity, high genetic structure and presence of inbreeding in this species. This fact should be taken into account in management when translocating seeds or plants from one site to another, due to possible processes of local adaptation that could preclude the optimum development of plants outside of their populations of origin (Habel et al., 2015). We perceive this as a weakness of the species, if the forms of management move to more intensive practices, which is highly probable in the light of the recent denomination of origin of raicilla (Diario Oficial de la Federación, 2019). However, recovery of this situation is possible based on promotion of these forestry systems that still maintain *A. maximiliana*.

Our research approach had restrictions related to the identification of local adaptation processes and phenotypic plasticity. As a result, we cannot make direct conclusions about the extent to which the wild and managed populations (either *in situ* or monocultures) of *A. maximiliana* are locally adapted. In addition, adoption of new approaches regarding developmental plasticity, variation in quantitative trait loci related to natural and artificial selection or niche construction theory (Chen et al., 2017; Piperno, 2017) could complete the understanding of diversification *versus* domestication contexts (Pickersgill, 2018). Despite this, we consider that the general patterns of intraspecific diversity in *in situ* contexts of management practices are still poorly documented, and that this study constitutes a valuable base on which to establish more specific research questions to further the understanding of the origin of domestication processes.

Finally, we recognize in this case study that, beyond the improvement of technical options to multiply the number of plants available per year, it would be more meaningful to consider broader aspects of sustainability and conservation. This would generate benefits not only for the raicilla production chain, but also for the range of environmental goods and services their habitat provides.

## Data Availability Statement

The datasets generated and analyzed for this study can be found in the Mendeley Data, http://dx.doi.org/10.17632/3379nnccmy.4.

## Author Contributions

DC-T and OV-P conceived and designed the study, partially organized fieldwork and carried out some final statistical analysis. SA-R and LV-S carried out the collection and first analysis of phenotypic and genetic data, respectively, as part of their bachelor theses. JP-A gave input on the study design and carried out some final statistical analysis and design of figures. JM-S led laboratory work. OH-G described the management practices which were the basis of the hypothesis of this study, he also partially organized fieldwork. All authors reviewed and approved the final manuscript.

## Conflict of Interest

The authors declare that the research was conducted in the absence of any commercial or financial relationships that could be construed as a potential conflict of interest.

## Footnotes

1 https://worldclim.org/version2
