# Supplementary material for: Morphological and Genetic Variation in Monocultures, Forestry Systems and Wild Populations of Agave maximiliana of Western Mexico: Implications for Its Conservation
Source: Front Plant Sci. 2020 Jun 17;11:817. doi: 10.3389/fpls.2020.00817 (PMC7313679; doi:10.3389/fpls.2020.00817)
Supplement: Supplementary file 1 [file Table_1.DOCX]

**Supplementary material SM1.** Native tree species associated with the studied populations and synthesis of management practices documented in Huerta-Galván (2018). PQF=*Pinus-Quercus* Forest, QPF=*Quercus-Pinus* Forest, QF=*Quercus* Forest. * 3-6 plants with the inflorescence removed were observed in all wild populations. **Laboratory of culture media and greenhouse are financed by an association between CBTA and CMPR. CBTA, Technological High School of Agriculture, for its Spanish acronym, located at Mascota, Jalisco. CMPR, Mexican Raicilla Promotion Council, for its Spanish acronym.

| Population | Code | Vegetation type and associated native tree species | Management practices |
| --- | --- | --- | --- |
| *Cultivated* |  |  |  |
| Cimarrón Chico | CC | None | Seeds are collected from plants growing in the forest situated within a linear distance of 6 km. Seedlings are looked after for 2-3 years with removal of weeds. The monoculture is watered during the dry season. Avocados and guava plants are also grown with the agave plants. |
| El Mosco | EM | None | Seeds are collected from plants growing in the forest situated within a linear distance of 7 km. For this, the biggest plants and those with the highest leaf sugar content are selected. Seeds are germinated using *in vitro* culture media, where 2 to 10 seedlings are obtained from each seed. Seedlings are then watered, grown and protected in a greenhouse property of CBTA-CMPR** for three years, until introduction to the monoculture, where weeds are eliminated with agrochemicals. |
| Rincón Seco | RS | None | Plants brought to CBTA. Weeds are eliminated with agrochemicals. |
| San Miguel | SM | None | Seeds are collected from plants growing in the forest situated within a linear distance of 2 km. Seedlings are looked after for 2 years. Weeds are eliminated with agrochemicals. |
| Las Palmas | LP | None | Seeds are collected from plants growing in the forest situated within a linear distance of 3 km. |
| *Managed* |  |  |  |
| Chiquilistlán | CH | PQF*: Pinus lumholtzii* B.L. Rob. & Fernald*, Quercus resinosa*  Liebm.  *Arctostaphylos pungens* Kunth, *Vachellia pennatula* (Schltdl. & Cham.) Seigler & Ebinger | Given the spatial location of the plants and low density of shrubs, we infer that this population is “tolerated”. This is, people let the agave plants naturally established in the forest stand and remove vegetation to eliminate competition. We could not find any people linked to this population. |
| La Berenjena | LB | QF*: Quercus magnoliifolia* Née, *Q. eduardi* Trel.*, Pinus devoniana* Lindl*., Arbutus xalapensis* Kunth | Owner increases plant density (“promotion”) with seeds collected from plants growing in the forest within a linear distance of 1.5 km. Seeds are germinated within the population and seedlings are then cared for in the backyard for 2 years, protected by shade mesh. Weeds are removed manually. |
| La Vieja | LV | PQF*: Pinus oocarpa*  Schiede ex Schltdl.*, P. luzmariae* Pérez de la Rosa*, P. lumholtzii, P. devoniana, Quercus magnoliifolia, Q. obtusata* Bonpl *Arbutus xalapensis, Juniperus durangensis* Martínez | Tolerated population. Only mature plants are extracted. Some plants are allowed to stand in order to form the inflorescence and allow natural regeneration of the populations. Bans are put in place when insufficient mature plants are identified. |
| Los Hornos | LH | PQF*: Pinus jaliscana*  Pérez de la Rosa*, P. devoniana, Podocarpus matudae*  Lundell*, Quercus calophylla* Schltdl. & Cham.*, Q. resinosa* Liebm.*, Q. mexiae*  L.M.González*, Q. castanea* Née*, Q. aff. aristata* Hook*.* & Arn*, Cecropia obtusifolia* Bertol*., Magnolia pacifica* A. Vázquez*, Clethra rosei* Britton. | Simple gathering is conducted only occasionally, since access to this population requires a 6-7 km hike. No other management practice is conducted. |
| Puerto la Campana | PC | PQF*: Pinus lumholtzii*, *P. oocarpa,, P. douglasiana* Martínez*, P. devoniana,*  *Eysenhardtia polystachya* (Ortega) Sarg.*, Quercus eduardi* Trel., *Q. obtusata, Q. rugosa* Née*, Q. candicans* Née,  *Arbutus glandulosa* Mart. & Gal., *Bejaria aestuans* Mutis ex L., *Vaccinium stenophyllum* Steud. *Vachellia pennatula* | Tolerated population. Only matured plants are extracted. Some plants are allowed to stand in order to form the inflorescence and allow natural regeneration of the populations. Bans are put in place when insufficient mature plants are identified. |
| Rincón de Mirandillas | RM | PQF*: Pinus lumholtzii, P. oocarpa, Quercus obtusata, Q. rugosa, Vachellia pennatula, Eysenhardtia polystachya, Verbesina sphaerocephala* A. Gray, *Baccharis* sp | Owner increases plant density (“promotion”) intensively, using seeds collected from plants growing in the forest within a linear distance of 5 km. Seeds are germinated and seedlings are looked after for 3 years in the backyard protected by shade mesh. Later, in the forest, weeds are eliminated using agrochemicals. |
| Sol de Oros | SO | PQF*: Pinus oocarpa, P. devoniana, Eysenhardtia polystachya, Quercus obtusata, Q. rugosa, Vachellia pennatula* | Owner increases agave plant density (“promotion”) together with pines. *Agave* seeds are collected from plants growing in the forest within a linear distance of 5 km. This producer also brought plants from the property of San Miguel. Seeds are germinated within the population and the resulting seedlings are then cared for the next 2 years. |
| *Wild** |  |  |  |
| El Nayar | EN | *Pinus lumholtzii, P.oocarpa,*  *P.montezumae* Lamb.*, P.douglasiana,Quercus resinosa, Q. eduardi, Vachellia pennatula* | - |
| El Palmito | EP | PQF: *Pinus lumholtzii, P.montezumae, Quercus gentryi* C.H. Mull*., Q. rugosa, Vachellia pennatula* | - |
| El Teúl | ET | QF*: Quercus deserticola* Trel.*, Q.eduardi, Prunus serotina* Ehrh.*, Juniperus blancoi* Martínez, *Vachellia pennatula, Bursera fagaroides* (Kunth) Engl. | - |
| Canelas | CN | QPF*: Quercus candicans* Née*, Q. castanea, Q. gentry, Q. jonesii* Trel.*, Q. magnoliifolia, Q. viminea* Trel.*, Bocconia arborea* S. Watson*, Pinus durangensis* Martínez*, P. oocarpa, P. maximinoi* H.E. Moore*, Juniperus deppeana* Steud*., J. flaccida* Schltdl*., Arbutus madre sis Prunus serotina, Urera baccifera* (L.) Gaudich | - |
| La Toma | LT | QPF*:Quercus resinosa, Q. gentry, Q. castanea, Pinus oocarpa, P.lumholtzii, Arbutus xalapensis, A. glandulosa* M. Martens & Galeotti | - |
| Valparaíso | VP | QPF: *Quercus eduardi, Q.resinosa, Pinus lumholtzii, Arctostaphylos pungens* | - |
| El Carrizo | EC | QPF *Pinus lumholtzii*, *P. douglasiana*, *P. durangensis*, *Quercus obtusata*, *Q. convallata* Trel., *Q. viminea*, *Juniperus flaccida*, *Arbutus* sp., *Garrya laurifolia* Hartw. ex Benth., *Clethra hartwegii* Britton, *Lippia* sp. | - |
