# Supplementary material for: Morphological and Genetic Variation in Monocultures, Forestry Systems and Wild Populations of Agave maximiliana of Western Mexico: Implications for Its Conservation
Source: Front Plant Sci. 2020 Jun 17;11:817. doi: 10.3389/fpls.2020.00817 (PMC7313679; doi:10.3389/fpls.2020.00817)
Supplement: Supplementary file 2 [file Table_2.DOCX]

**Supplementary material SM2.** Pearson correlation matrix of 16 morphological traits. Bold numbers and codes correspond a high collinearity with r ≥ 0.9. Codes: TPH=Total plant height, MinD=Minimum diameter, MxD=Maximum diameter, LL=Leaf length, LWm=Leaf width at middle, MxLW=Maximum leaf width, TTL=Terminal thorn length, TTW=Terminal thorn width at the base, NT=Number of teeth, NT10=Number of teeth in 10 cm^2^, LTL=Longest tooth length, NT/LL=Number of teeth/ leaf length (thorniness), NT10/LL=Number of teeth in 10cm/ leaf length (spacing), LWm/MxLW=Leaf width at middle / Maximum leaf width (width index), LL/MxLW=Leaf length / Maximum leaf width (leaf shape),TTW/TTL=Terminal thorn width at the base / Terminal thorn length (thorn shape).

|  | TPH | MinD | MxD | LL | **LWm** | MxLW | TTL | TTW | NT | **NT10** | LTL | NT/LL | NT10/LL | LWm/MxLW | LL/ MxLW | TTW/TTL |
| --- | --- | --- | --- | --- | --- | --- | --- | --- | --- | --- | --- | --- | --- | --- | --- | --- |
| TPH |  |  |  |  |  |  |  |  |  |  |  |  |  |  |  |  |
| MinD | 0.848 |  |  |  |  |  |  |  |  |  |  |  |  |  |  |  |
| MxD | 0.855 | **0.975** |  |  |  |  |  |  |  |  |  |  |  |  |  |  |
| LL | **0.919** | **0.933** | **0.941** |  |  |  |  |  |  |  |  |  |  |  |  |  |
| LWm | 0.776 | 0.758 | 0.740 | 0.804 |  |  |  |  |  |  |  |  |  |  |  |  |
| MxLW | 0.747 | 0.726 | 0.708 | 0.772 | **0.972** |  |  |  |  |  |  |  |  |  |  |  |
| TTL | 0.271 | 0.219 | 0.237 | 0.295 | 0.266 | 0.250 |  |  |  |  |  |  |  |  |  |  |
| TTW | -0.055 | 0.008 | 0.005 | -0.062 | 0.057 | 0.006 | 0.329 |  |  |  |  |  |  |  |  |  |
| NT | 0.493 | 0.403 | 0.394 | 0.449 | 0.285 | 0.312 | 0.172 | -0.159 |  |  |  |  |  |  |  |  |
| NT10 | -0.096 | -0.242 | -0.254 | -0.220 | -0.223 | -0.176 | 0.008 | -0.132 | 0.687 |  |  |  |  |  |  |  |
| LTL | 0.210 | 0.128 | 0.114 | 0.128 | 0.316 | 0.261 | 0.309 | 0.187 | -0.030 | -0.093 |  |  |  |  |  |  |
| NT/LL | -0.160 | -0.287 | -0.297 | -0.270 | -0.295 | -0.246 | -0.081 | -0.166 | 0.698 | **0.924** | -0.146 |  |  |  |  |  |
| NT10/LL | -0.533 | -0.651 | -0.654 | -0.657 | -0.580 | -0.535 | -0.206 | -0.095 | 0.184 | 0.785 | -0.155 | 0.793 |  |  |  |  |
| LWm/MxLW | 0.149 | 0.128 | 0.126 | 0.151 | 0.222 | 0.438 | 0.052 | -0.186 | 0.192 | 0.096 | -0.105 | 0.078 | -0.025 |  |  |  |
| LL/ MxLW | 0.402 | 0.452 | 0.486 | 0.501 | -0.088 | -0.102 | 0.089 | -0.200 | 0.319 | -0.043 | -0.281 | -0.031 | -0.274 | -0.081 |  |  |
| TTW/TTL | -0.219 | -0.145 | -0.160 | -0.255 | -0.111 | -0.138 | -0.456 | 0.663 | -0.255 | -0.122 | -0.032 | -0.080 | 0.075 | -0.164 | -0.263 |  |
