# Supplementary material for: Morphological and Genetic Variation in Monocultures, Forestry Systems and Wild Populations of Agave maximiliana of Western Mexico: Implications for Its Conservation
Source: Front Plant Sci. 2020 Jun 17;11:817. doi: 10.3389/fpls.2020.00817 (PMC7313679; doi:10.3389/fpls.2020.00817)
Supplement: Supplementary file 3 [file Table_3.docx]

| Locus | | Primer sequence (5´-3´) | Repeated motive | Alignment temperature (°C) | Literature |
| --- | --- | --- | --- | --- | --- |
| 1763 | F:CGATCGAGGAGCTGAAGTCT  R: TTCTTCAGGGATGAGCCATT | | (AGA)_15_ | 61 | Félix-Valdez et al., 2016 |
| 2-12 | F: CGTCACCGCCAGTTAAGAG  R: GTGCCCATGATCTGTGGTTG | | (CT)_7_ | 60.5 | Lindsay  et al*.*, 2012 |
| C20 | F:AGTTGCTGAAATCGAAGATCCG  R: TCTCGGTTGGATTCGGTCG | | (AG)_7_ | 57.9 | Lindsay  et al., 2012 |
| C28 | F: CCTCCTGCGTAGAGGAACC  R: TCTCCGTTTGAACTCCGTG | | (AAG)_4_ | 56.9 | Lindsay  et al., 2012 |
| C35 | F: TGTGTGTTTAGCAGTGCCG  R:AGCGGATCTGAGCAAAATGAG | | (ATT)_4_ | 58.8° | Lindsay  et al., 2012 |
| 311 | F: TGTGGCGGCTAAAAGAAGG  R: CCGATCCGGCGTAATTCTC | | (AAC)_4_ | 61.6 | Lindsay  et al., 2012 |
| 1448 | F: GTGCGGGCTCACTTATGTTT  R: CAAACCAAAACCAGATCAAAGTC | | (AG)_28_ | 62.8 | LANGEBIO |
| 7a | Not published | | (CT)_4_…(CT)_4_..  (CT)_7_..(CT)_16_ | 57.6 | University of Georgia |
| 8D | Not published | | (CT)_7_ | 62.8 | University of Georgia |

**Supplementary material SM3**. Primer used in the genetic analysis of wild, manage and cultivated populations of *A. maximiliana.*
