# Supplementary material for: Morphological and Genetic Variation in Monocultures, Forestry Systems and Wild Populations of Agave maximiliana of Western Mexico: Implications for Its Conservation
Source: Front Plant Sci. 2020 Jun 17;11:817. doi: 10.3389/fpls.2020.00817 (PMC7313679; doi:10.3389/fpls.2020.00817)
Supplement: Supplementary file 4 [file Table_4.DOCX]

**Supplementary material SM4.** Genetic and morphological diversity in all cultivated, managed and wild populations of *A. maximiliana*. N: sample size; P: percentage of polymorphic loci; A: number of alleles per locus; A_E_: effective number of alleles per locus; H_O_: observed heterozygosity; H_E_: expected heterozygosity in Hardy-Weinberg equilibrium; IMD: index of morphological diversity ± standard error.

| Population | N | P | A | A_E_ | H_O_ | H_E_ | IMD |
| --- | --- | --- | --- | --- | --- | --- | --- |
| *Cultivated* |  |  |  |  |  |  |  |
| CC | 12.778 | 100 | 2.333 | 1.978 | 0.466 | 0.476 | 0.620 |
|  | ±0.703 | - | ±0.236 | ±0.152 | ±0.122 | ±0.032 | ±0.080 |
| EM | 11.222 | 100 | 2.444 | 2.060 | 0.360 | 0.498 | 0.673 |
|  | ±0.641 | - | ±0.176 | ±0.128 | ±0.076 | ±0.034 | ±0.061 |
| RS | 12.778 | 88.89 | 2.333 | 1.841 | 0.340 | 0.396 | 0.653 |
|  | ±0.741 | - | ±0.289 | ±0.194 | ±0.132 | ±0.075 | ±0.127 |
| LP | 13.000 | 88.89 | 2.111 | 1.690 | 0.271 | 0.339 | - |
|  | ±0.624 | - | ±0.200 | ±0.195 | ±0.116 | ±0.077 |  |
| Mean | 12.445 | 94.44 | 2.305 | 1.892 | 0.359 | 0.427 | 0.648 |
|  | ±0.822 | ±6.41 | ±0.140 | ±0.162 | ±0.081 | ±0.0739 | ±0.027 |
| *Managed* |  |  |  |  |  |  |  |
| CH | 13.000 | 100 | 2.222 | 1.508 | 0.185 | 0.293 | 0.700 |
|  | ±0.441 | - | ±0.147 | ±0.150 | ±0.037 | ±0.058 | ±0.053 |
| LB | 13.889 | 100 | 2.889 | 2.163 | 0.267 | 0.503 | 0.747 |
|  | ±0.423 | - | ±0.309 | ±0.205 | ±0.048 | ±0.046 | ±0.046 |
| LV | 11.000 | 100 | 2.111 | 1.714 | 0.209 | 0.376 | 0.493 |
|  | ±1.014 | - | ±0.111 | ±0.167 | ±0.046 | ±0.054 | ±0.160 |
| LH | 13.222 | 100 | 2.333 | 1.803 | 0.283 | 0.397 | 0.720 |
|  | ±0.324 | - | ±0.236 | ±0.213 | ±0.062 | ±0.055 | ±0.020 |
| PC | 13.556 | 66.67 | 2.111 | 1.934 | 0.367 | 0.366 | 0.693 |
|  | ±0.530 | - | ±0.351 | ±0.302 | ±0.131 | ±0.099 | ±0.099 |
| RM | 11.778 | 88.89 | 2.333 | 1.830 | 0.178 | 0.393 | 0.693 |
|  | ±0.760 | - | ±0.236 | ±0.192 | ±0.056 | ±0.075 | ±0.030 |
| Mean | 12.741 | 92.59 | 2.33 | 1.825 | 0.248 | 0.388 | 0.674 |
|  | ±1.127 | ±13.45 | ±0.290 | ±0.219 | ±0.072 | ±0.0678 | ±0.091 |
| *Wild* |  |  |  |  |  |  |  |
| ET | 13.222 | 100 | 2.222 | 1.757 | 0.258 | 0.371 | 0.640 |
|  | ±0.596 | - | ±0.147 | ±0.197 | ±0.103 | ±0.070 | ±0.020 |
| EN | 12.222 | 100 | 2.556 | 2.050 | 0.291 | 0.496 | 0.480 |
|  | 0.572 | - | ±0.242 | ±0.140 | ±0.078 | ±0.031 | ±0.072 |
| EC | 11.111 | 100 | 2.778 | 2.097 | 0.402 | 0.502 | - |
|  | ±1.195 | - | ±0.278 | ±0.156 | ±0.096 | ±0.037 |  |
| EP | 13.444 | 88.89 | 2.444 | 1.765 | 0.157 | 0.385 | 0.653 |
|  | ±0.338 |  | ±0.294 | ±0.175 | ±0.044 | ±0.065 | ±0.080 |
| Mean | 12.50 | 97.22 | 2.5 | 1.917 | 0.277 | 0.438 | 0.591 |
|  | ±1.067 | ±5.55 | ±0.231 | ±0.181 | ±0.1 | ±0.07 | ±0.096 |
