# Supplementary material for: Morphological and Genetic Variation in Monocultures, Forestry Systems and Wild Populations of Agave maximiliana of Western Mexico: Implications for Its Conservation
Source: Front Plant Sci. 2020 Jun 17;11:817. doi: 10.3389/fpls.2020.00817 (PMC7313679; doi:10.3389/fpls.2020.00817)
Supplement: Supplementary file 5 [file Table_5.DOCX]

**Supplementary material SM5.** First and second discriminant functions explaining 70% of the variation dispersion. LD1: first function (49%); LD2: second function (21%). The variables with highest values are shown in bold font. Codes: TPH=Total plant height, MxD=Maximum diameter, MxLW=Maximum leaf width, TTL=Terminal thorn length, TTW=Terminal thorn width at the base, NT=Number of teeth, LTL=Longest tooth length, NT/LL=Number of teeth/ leaf length (thorniness), NT10/LL=Number of teeth in 10cm/ leaf length (spacing), LWm/MxLW=Leaf width at middle / Maximum leaf width (width index), LL/MxLW=Leaf length / Maximum leaf width (leaf shape),TTW/TTL=Terminal thorn width at the base / Terminal thorn length (thorn shape).

| Trait | LD1 | LD2 | Trait |
| --- | --- | --- | --- |
| MxD | 0.22186317 | **2.2445636** | **NT/LL** |
| NT/LL | 0.20802344 | **1.6574078** | **MxD** |
| TTW/TTL | 0.13280584 | 0.8681883 | MxLW |
| TTW | 0.09581605 | 0.5381412 | LL/MxLW |
| TTL | -0.01096317 | 0.4842079 | TTW |
| LTL | -0.03786449 | 0.1498407 | LTL |
| TPH | -0.05809464 | -0.1557444 | TTW/TTL |
| NT10/LL | -0.23570014 | -0.2326322 | LWm/MxLW |
| NT | -0.26377689 | -0.6326683 | TTL |
| LWm/MxLW | -0.50212239 | -0.7975296 | TPH |
| LL/MxLW | -0.74984027 | -0.9589677 | NT10/LL |
| **MxLW** | **-2.27363725** | **-2.9862883** | **NT** |
