# Supplementary material for: Morphological and Genetic Variation in Monocultures, Forestry Systems and Wild Populations of Agave maximiliana of Western Mexico: Implications for Its Conservation
Source: Front Plant Sci. 2020 Jun 17;11:817. doi: 10.3389/fpls.2020.00817 (PMC7313679; doi:10.3389/fpls.2020.00817)
Supplement: Supplementary file 6 [file Table_6.DOCX]

**Supplementary material SM6.** Means and coefficient of variation (bold numbers) of 12 morphological traits evaluated on the 17 populations of *Agave maximiliana* in Western Mexico. Code of population: CC=Cimarrón Chico, EM=El Mosco, RS=Rincón Seco, SM=San Miguel, LP=Las Palmas, CH=Chiquilistlán, LB=La Berenjena, LV=La Vieja, LH=Los Hornos, PC=Puerto la Campana, RM=Rincón de Mirandillas, SO=Sol de Oros, EN=El Nayar, EP=El Palmito, ET=El Teúl, CN=Canelas, LT=La Toma, VP=Valparaíso, EC=El Carrizo. Code of morphological trait: TPH=Total plant height, MxD=Maximum diameter, MxLW=Maximum leaf width, TTL=Terminal thorn length, TTW=Terminal thorn width at the base, NT=Number of teeth, LTL=Longest tooth length, NT/LL=Number of teeth/ leaf length (thorniness), NT10/LL=Number of teeth in 10cm/ leaf length (spacing), LWm/MxLW=Leaf width at middle / Maximum leaf width (width index), LL/MxLW=Leaf length / Maximum leaf width (leaf shape),TTW/TTL=Terminal thorn width at the base / Terminal thorn length (thorn shape).

| TRAIT | TPH | | MxD | | LWm | | TTL | | TTW | | NT | | LTL | | NT/LL | | NT10/LL | | LWm/MxLW | | LL/ MxLW | | TTW/TTL | |
| --- | --- | --- | --- | --- | --- | --- | --- | --- | --- | --- | --- | --- | --- | --- | --- | --- | --- | --- | --- | --- | --- | --- | --- | --- |
| POP | Mean | VC | Mean | VC | Mean | VC | Mean | VC | Mean | VC | Mean | VC | Mean | VC | Mean | VC | Mean | VC | Mean | VC | Mean | VC | Mean | VC |
| **Cultivated** |  | |  |  |  |  |  |  |  |  |  |  |  |  |  |  |  |  |  |  |  |  |  |  |
| CC | 120.5 | **11.7** | 175.8 | **10.2** | 20.3 | **14.8** | 33.7 | **20.4** | 4.3 | **19.1** | 53.9 | **24.0** | 9.7 | **28.2** | 0.6 | **26.9** | 0.1 | **48.5** | 0.9 | **4.9** | 4.6 | **18.5** | 0.1 | **19.4** |
| EM | 117.2 | **14.5** | 212.3 | **14.1** | 19.6 | **11.7** | 31.7 | **16.6** | 4.8 | **16.4** | 78.9 | **23.6** | 8.7 | **16.1** | 0.8 | **20.2** | 0.1 | **49.4** | 0.9 | **7.6** | 5.0 | **13.1** | 0.2 | **18.9** |
| RS | 86.5 | **11.8** | 149.0 | **14.0** | 18.1 | **11.0** | 35.4 | **12.9** | 3.6 | **14.0** | 73.6 | **41.5** | 8.3 | **16.8** | 1.0 | **44.2** | 0.1 | **50.6** | 0.9 | **5.0** | 4.3 | **17.5** | 0.1 | **17.6** |
| SM | 82.1 | **25.1** | 159.5 | **18.6** | 16.4 | **17.5** | 31.9 | **22.9** | 5.1 | **31.3** | 50.6 | **21.2** | 8.2 | **19.8** | 0.7 | **29.4** | 0.1 | **49.2** | 0.8 | **4.2** | 4.4 | **18.4** | 0.2 | **17.8** |
| **Managed** |  | |  |  |  |  |  |  |  |  |  |  |  |  |  |  |  |  |  |  |  |  |  |  |
| CH | 119.9 | **11.8** | 203.4 | **17.0** | 26.0 | **12.0** | 33.6 | **17.0** | 4.2 | **12.8** | 67.6 | **37.2** | 11.3 | **13.7** | 0.7 | **36.8** | 0.1 | **46.9** | 0.9 | **6.1** | 3.9 | **15.1** | 0.1 | **19.4** |
| LB | 116.1 | **14.2** | 218.0 | **16.3** | 19.5 | **14.9** | 28.0 | **30.6** | 4.0 | **23.6** | 88.6 | **34.6** | 8.0 | **25.3** | 0.9 | **34.4** | 0.1 | **49.0** | 0.9 | **6.6** | 5.2 | **10.8** | 0.1 | **31.5** |
| LV | 70.6 | **21.5** | 135.2 | **11.6** | 14.9 | **8.6** | 30.2 | **17.6** | 5.4 | **24.2** | 49.1 | **34.2** | 10.1 | **20.0** | 0.8 | **21.9** | 0.2 | **24.0** | 0.8 | **4.2** | 3.9 | **18.5** | 0.2 | **18.3** |
| LH | 110.0 | **22.2** | 211.3 | **14.3** | 22.0 | **13.7** | 31.8 | **13.9** | 3.7 | **16.4** | 76.9 | **41.7** | 8.0 | **24.0** | 0.8 | **40.4** | 0.1 | **48.5** | 0.9 | **6.3** | 4.6 | **21.7** | 0.1 | **12.7** |
| PC | 65.6 | **24.4** | 131.5 | **22.7** | 14.2 | **23.1** | 29.6 | **21.1** | 5.2 | **18.3** | 41.1 | **46.2** | 8.1 | **17.9** | 0.8 | **47.4** | 0.2 | **75.0** | 0.9 | **7.4** | 3.8 | **20.7** | 0.2 | **16.6** |
| RM | 77.5 | **19.3** | 153.8 | **15.5** | 14.9 | **18.6** | 29.5 | **17.0** | 5.1 | **28.6** | 60.5 | **36.0** | 8.3 | **29.7** | 0.9 | **24.6** | 0.2 | **31.2** | 0.8 | **5.7** | 4.5 | **19.2** | 0.2 | **21.0** |
| SO | 118.9 | **16.9** | 217.3 | **8.9** | 26.7 | **17.4** | 30.9 | **33.4** | 5.8 | **34.9** | 45.2 | **28.0** | 11.4 | **16.0** | 0.5 | **32.9** | 0.0 | **44.1** | 0.9 | **5.2** | 3.8 | **13.6** | 0.2 | **36.3** |
| **Wild** |  |  |  |  |  |  |  |  |  |  |  |  |  |  |  |  |  |  |  |  |  |  |  |  |
| EN | 72.5 | **15.6** | 130.6 | **6.2** | 14.7 | **7.1** | 28.9 | **9.9** | 4.7 | **14.8** | 52.0 | **21.6** | 10.6 | **22.3** | 0.9 | **22.5** | 0.2 | **29.4** | 0.9 | **11.0** | 3.8 | **9.3** | 0.2 | **15.7** |
| EP | 95.9 | **22.1** | 145.7 | **21.2** | 20.0 | **11.5** | 35.1 | **15.5** | 4.3 | **24.3** | 90.3 | **16.8** | 9.6 | **23.4** | 1.3 | **13.5** | 0.2 | **30.3** | 0.9 | **7.5** | 3.6 | **8.9** | 0.1 | **14.1** |
| ET | 58.3 | **14.1** | 101.3 | **18.5** | 10.8 | **15.9** | 29.8 | **21.5** | 4.3 | **23.8** | 69.9 | **31.5** | 8.2 | **19.5** | 1.5 | **25.7** | 0.4 | **40.0** | 0.8 | **5.9** | 4.3 | **13.9** | 0.1 | **28.0** |
| CN | 128.4 | **10.2** | 192.9 | **15.4** | 21.3 | **8.0** | 38.0 | **20.2** | 4.8 | **12.9** | 142.0 | **21.5** | 11.1 | **17.5** | 1.5 | **20.3** | 0.2 | **25.6** | 0.9 | **5.3** | 4.4 | **15.3** | 0.1 | **22.8** |
| LT | 68.4 | **24.3** | 118.5 | **25.0** | 12.1 | **16.9** | 28.5 | **12.1** | 4.0 | **26.4** | 48.8 | **19.1** | 9.1 | **14.8** | 1.0 | **15.7** | 0.2 | **29.5** | 0.8 | **8.3** | 4.3 | **16.9** | 0.1 | **23.3** |
| VP | 59.5 | **9.4** | 91.1 | **16.6** | 11.0 | **17.9** | 27.4 | **19.7** | 5.0 | **23.8** | 32.0 | **26.7** | 9.7 | **18.5** | 0.8 | **29.2** | 0.2 | **45.9** | 0.8 | **2.8** | 3.6 | **14.4** | 0.2 | **24.6** |
